# Pea-Wheat Rotation Affects Soil Microbiota Diversity, Community Structure, and Soilborne Pathogens

**DOI:** 10.3390/microorganisms10020370

**Published:** 2022-02-05

**Authors:** Sheridan Lois Woo, Francesca De Filippis, Maurizio Zotti, Albert Vandenberg, Pierre Hucl, Giuliano Bonanomi

**Affiliations:** 1 Department of Pharmacy, University of Naples Federico II, Via Domenico Montesano, 49, 80131 Napoli, Italy; 2 Task Force on Microbiome Studies, University of Naples Federico II, 80131 Napoli, Italy; francesca.defilippis@unina.it (F.D.F.); giuliano.bonanomi@unina.it (G.B.); 3 Department of Agricultural Sciences, University of Naples Federico II, Via Università 100, 80055 Portici, Italy; maurizio.zotti@unina.it; 4 Department of Plant Sciences, University of Saskatchewan, Saskatoon, SK S7N5A8, Canada; bert.vandenberg@usask.ca (A.V.); pierre.hucl@usask.ca (P.H.)

**Keywords:** soil microbiota, beneficial microbes, soilborne pathogens, *Fusarium graminearum*, microbial diversity

## Abstract

Intensive cultivation based on monocultures has a significant impact on ecosystem function, and sustainable agriculture must rely on alternative methods, including crop rotation. On the Canadian prairies, the use of pulse crops is a common practice, but few studies have investigated the impact on soil microorganisms. Here, we studied the effect of pea, wheat, pea–wheat rotation, and fallow in bulk soil bacterial and fungal communities. We characterized soil microbiota by high-throughput sequencing of 16S and 18S rRNA genes for bacteria and eukaryotes. Different crop rotations and fallow significantly modified soil community composition, as well as bacterial and fungal diversity. Pea alone caused a strong reduction of bacterial and fungal richness and diversity compared to wheat, pea–wheat rotation, and fallow. Notably, pea–wheat rotation increased the abundance of *Fusarium graminearum* compared to other management practices. The bacterial community was less responsive to crop rotation identity compared to the fungal microbiota, and we found minor differences at the phylum level, with an increase in Actinobacteria in fallow and Firmicutes in wheat. In summary, our study demonstrated that rotations alter bulk soil microbial community diversity and composition in Canadian prairies. The frequent use of pea in rotation with wheat should be carefully evaluated, balancing their ecological effects on nitrogen mineralization, water conservation, and impact on beneficial, as well as pathotrophic, fungi.

## 1. Introduction

Intensive cultivation has a significant impact on the overall ecosystem due to the large inputs of irrigation water, fertilizers, and agrochemicals required for maintaining crop productivity [1]. However, modern and sustainable agriculture must implement production systems that reduce external energy inputs without penalizing crop yield. In this view, farming systems that rely on crop rotation to improve plant nutrition and control weeds, pests, and plant pathogens is an old but attractive and environmentally friendly approach [2].

Crop rotation is an ancient method used to overcome soil sickness (SS), i.e., the rise of negative soil conditions for crop growth induced by monoculture [3]. The beneficial effects of crop rotation include the improvement of soil physical structure and aggregation [4], the increasing diversity of soil microbiota and associated beneficial microbes [5], and the control of soilborne, as well as airborne, pathogens by breaking their natural life cycle [6,7,8]. In fact, crop rotation alleviates SS by decreasing pathogen inoculum [9,10,11], as well as by reducing the deleterious effect of autotoxic chemical compounds released during crop residue decomposition [12]. However, despite the agronomic advantages of crop rotation, the inclusion of such practice in the current cultivation management is challenging, especially in intensive agriculture systems.

Numerous studies have tried to understand the impact of crop rotation on microbiome structure and associated ecosystem functions. Microbial diversity, enzymatic activities, soil respiration, and organic carbon sequestration are generally higher under cultivations that correspond to monoculture or fallow [13,14,15]. For instance, Lupwayi and co-workers [16] found that pea and red clover rotations increased soil and rhizosphere bacterial diversity and functionality compared with continuous wheat cultivation. More recently, Borrell in 2017 [17] reported that the use of pea, lentil, and chickpea in rotation affects the fungal community of wheat, promoting the activity of arbuscular mycorrhizal fungi associated with wheat. Although a number of studies have addressed the impact of wheat-based crop rotations on the diversity of the soil microbiome, most of the studies focused either on bacterial or fungal communities, and there were few studies that studied the whole soil microbiome [18].

The use of pulse crops (e.g., lentil, pea, and chickpea) to enhance the rotation diversity in traditional cereal–fallow agricultural systems has become a popular alternative to summer fallow [19]. Pulses usually increase wheat yield [20], but the effect on the soil microbiome and wheat pathogens is controversial. For example, Niu et al. in 2018 [21] reported that the frequent use of pulses in the rotation increased the proportion of pathotrophic fungi, including *Botrytis cinerea*, *Fusarium solani*, and *F. graminearum*. In this context, the present study will investigate how long-term pea–wheat rotation can affect the soil bacterial and fungal microbiome. Specifically, by utilizing an ongoing long-term study established in Saskatoon (Canada), it was hypothesized that soils in a pea–wheat rotation could lead to higher microbiota diversity in comparison to the soil microbial communities from fields of monocultures or those managed by fallow. To test this hypothesis, the soil microbiota was characterized by high-throughput sequencing of 16S and 18S rRNA genes for bacteria and eukaryotes. Specifically, the aims of this study were to: (i) describe the differences in the microbial community of soils managed by a pea–wheat rotation, their separate individual monocultures, and fallow; (ii) explore the impact of a crop rotation system on the abundance of the principal pathogens of wheat; and (iii) test if pea–wheat rotation increases microbiota diversity compared to monocultures.

## 2. Materials and Methods

### 2.1. Study Site, Experimental Design, and Soil Sampling

The experiment was conducted at the University of Saskatchewan, Skarsgard Research Farm (52° N and 106° W), located about 22 km from Saskatoon SK, Canada. The study area is considered a cold semi-arid climate (Köppen type “BSk”) with a mean annual rainfall of 340.4 mm, mean annual temperature of 3.3 °C, and mean monthly temperatures ranging from 18.2 °C in August to −13.9 °C in January. The soil is an Elstow Orthic Dark Brown Loam containing 28% sand, 55% silt, and 17% clay; average soil pH was 6.6, and soil NO_3_-N to a depth of 20 cm was 33.5 kg/ha.

The experiment was a randomized complete block design consisting of 10 pea–wheat rotations in which the first season initiated with the pea cultivation: *Pisum sativum* L., cultivars Alfetta, CDC Golden, CDC Mozart, CDC Rocket, Delta, DS Admiral, Grande, Majoret, Reward, or Trapper. Subsequently, in the following year, hard red spring bread wheat (*Triticum aestivum* L. cv AC Barrie) was seeded into the pea stubble remaining from the previous year. Three other crop cultivations consisted of the pea cv CDC Golden alone and wheat AC Barrie alone, planted individually without the pea stubble, plus the no pea–no wheat fallow system. The individual replicated block was 3.6 m × 48 m for the stubble of the pea cultivars (10 cv x 3 reps), and the individual plot size for the pea–wheat crop rotation or single crop plantings was 1.6 × 3.6 m, whereby each crop cultivation consisted of three replicates.

One week before physiological maturity, soil samples were collected with a 2 cm diameter soil probe, to a depth of 20 cm, from each of the plots, then combined to obtain a bulk soil sample for each cultivation condition, from which aliquots were conserved in diverse screw-cap tubes for each of the 3 replicates. A total of 39 soil samples were collected from the field and maintained at 4 °C storage until further analysis. A portion of each fresh soil sample was used for culturing the microorganisms and determining the colony-forming units, then the remaining soil samples were conserved at −20 °C prior to DNA extraction.

### 2.2. Culturable Microorganisms, Soil Microbiota, and Biodiversity Assessment

From the soil samples collected in the field, a pool was prepared for each plot replicate consisting of 3 g from each of the 3 tubes collected, which was mixed to obtain 3 combined replicates for each pea–wheat cultivar cultivation. A 1 g aliquot was used for the quantification of the number of fungal and bacterial colony-forming units (CFU) in four conditions: fallow, pea only, wheat only, and in two pea-wheat rotations with DS Admiral-AC Barrie and Majoret-AC Barrie (based on the extremes in %NHI, highest and lowest, respectively; data not shown). Briefly, a soil suspension was prepared in a serial dilution from 10^−3^ to 10^−7^ with sterile water, then a 100 μL aliquot from each dilution series was transferred to the surface of 90 mm plates containing solid substrates Potato Dextrose Agar, or Rose Bengal Agar with Chloramphenicol (HiMedia Laboratories, Mumbai, India) for the fungi or Plate Count Agar (HiMedia Laboratories, Mumbai, India) for the bacteria, augmented with Igepal (Sigma; Milan). Soil suspensions were distributed uniformly on the substrate surface with an L-spreader and incubated at 25 °C. Every 24 h, the number of fungal or bacterial colonies was counted to determine the abundance of the microorganisms present for 5 days.

Microbial DNA was extracted from the soil samples using the Macherey-Nagel NucleoSpin Soil kit (Thermo-Fisher Scientific, Rodano, Italy) following the manufacturer’s instructions. From total DNA, V1–V3 regions of the 16S rRNA gene (about 520 bp) and a portion of the 18S rRNA gene (about 436 bp) were amplified. The 16S rRNA gene was amplified using primers Gray28F 5′-TTTGATCNTGGCTCAG and Gray519r 5′-GTNTTACNGCGGCKGCTG, while 18S-580f 5′-ATTCCAKCTCCAAKAGCG and 18S-997r 5′-GACTACGAYGGTATCTIATC were used for the 18S rRNA gene. PCR conditions were previously reported by Bonanomi et al. in 2016. After purification of the PCR products with the Agencourt AMPure beads (Beckman Coulter, Milan, Italy) and quantification by fluorometry, samples were sequenced on a GS Junior platform (454 Life Sciences, Roche Diagnostics, Italy), according to the manufacturer’s instructions. Raw sequences were deposited on the Sequence Read Archive (SRA) of the National Center for Biotechnology Information (NCBI), under Accession Number PRJNA800669.

### 2.3. Data Analysis

Raw reads were filtered and analyzed by using the QIIME 1.9.0 software [22]. Reads shorter than 300 bp, with more than 1 primer mismatch and with an average quality score lower than 25, were discarded. The taxonomic data obtained from the analysis of the fungi and bacteria identified were used to determine the distribution of the prevalent groups in the soil samples collected from the thirteen diverse cultivation systems. Only the taxonomic organisms with values > 0.5% of the total abundance were considered for the subsequent analysis.

Operational taxonomic units (OTUs) were picked through a de novo approach and the uclust method, and taxonomic assignment was obtained by using the RDP classifier and the Greengenes [23] or the Silva SSU/LSU rRNA gene database release 123 [24]. Chloroplast and Streptophyta contamination, as well as singletons, were removed, and the relative abundance of other taxa was recalculated. OTU tables were rarefied at the lowest number of sequences per sample.

From the OTU tables, alpha diversity indices were calculated to assess species richness in both bacterial and fungal communities. Indices used were number of OTUs (counts of different taxa), Chao1 diversity index (abundance-based index emphasizing the contribution of rarer taxa), and Shannon index (relating abundance and number of taxa). Principal component analysis (PCA) of the OTU tables were used to assess the association of specific taxa, within bacterial and fungal communities, to different crop rotation management systems. PCA was performed using Statistica 10 software.

The 16S and 18S rRNA gene sequences are available at the Sequence Read Archive (SRA) of the National Center for Biotechnology Information (NCBI).

## 3. Results

### 3.1. Crop Rotation Effects on the Culturable Microorganisms

**Figure 1:**
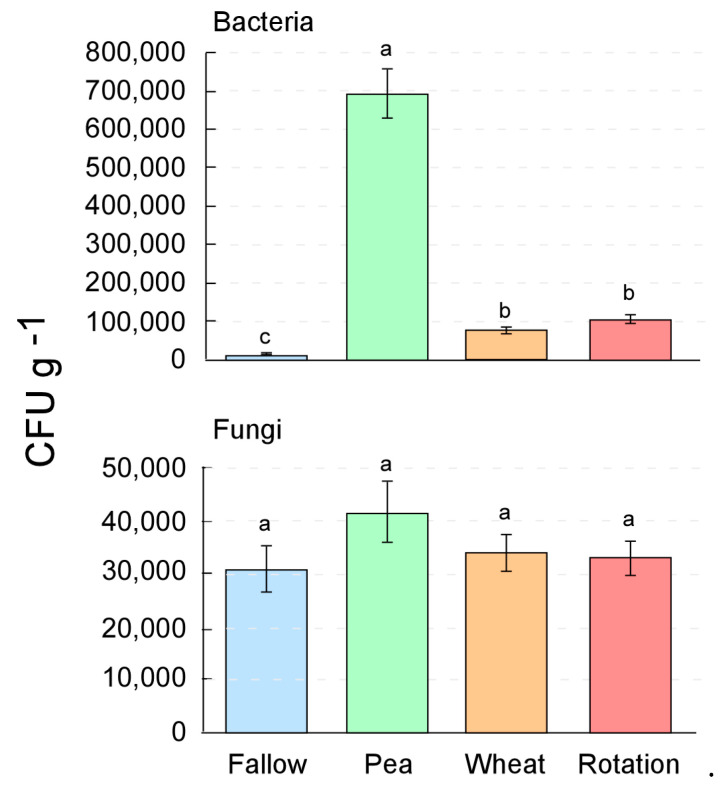
Number of bacterial (above) and fungal (below) colony-forming units (CFU per g of soil) in soils cultured on Plate Count Agar (bacteria) and Rose Bengal Agar containing Chloramphenicol (fungi) of samples obtained from diverse cultivations systems: fallow, pea only, wheat only, and pea–wheat rotation. Values are the average of three replicates, different letters indicate significant (*p* < 0.05) differences assigned according to univariate ANOVA and post-hoc Duncan test.

The number of bacterial CFU was highest in the pea-only crop system and lowest in the fallow system (Figure 1). The wheat alone was intermediate, whereas the bacteria determined in the wheat in rotation with the pea was slightly higher. The number of fungal colony-forming units of the soils originating from the selected cropping systems of fallow, pea only, wheat only, and the pea–wheat rotation was not significantly different, ranging from 1.17 to 2.43 × 10^4^ CFU per g soil (Figure 1).

### 3.2. Crop Rotation Effects on Microbial Diversity

**Figure 2:**
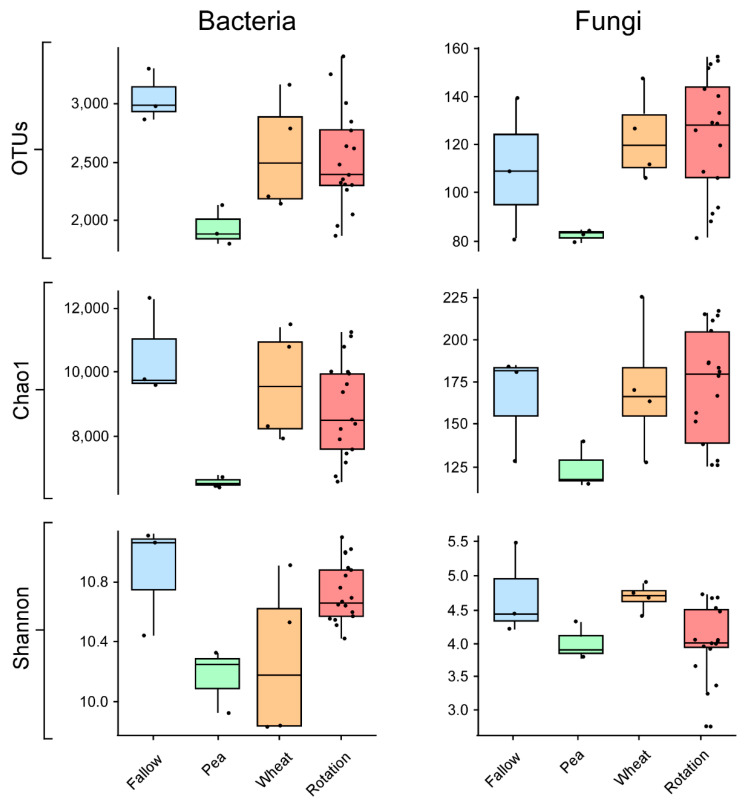
Box plots showing the number of observed OTUs, Shannon, and Chao1 diversity indices based on bacterial (**left** side) and eukaryotic (**right** side) communities in the soil samples subject to different crop cultivation systems: Fallow, pea only, wheat only, and pea–wheat rotation. Boxes represent the interquartile range (IQR) between the first and third quartiles, and the line inside represents the median (2nd quartile). Whiskers denote the lowest and the highest values within 1.5 × IQR from the first and third quartiles, respectively.

A total of 85,438 and 107,308 high-quality reads were analyzed, with an average length of 530 and 458 bp for Bacteria and Eukarya, respectively. Crop management practices showed a significant effect on microbial diversity, with a similar effect observed both in bacterial and fungal communities (Figure 2). Bacterial and fungal diversity and richness were always the lowest in pea monoculture. On the contrary, bacterial diversity was the highest in fallow, followed by wheat monoculture and pea–wheat rotation. As for fungi, wheat monoculture and rotation showed the highest richness and diversity, with fallow having slightly lower values (Figure 2).

### 3.3. Crop Rotation Effects on Microbiota Structure and Composition

**Figure 3:**
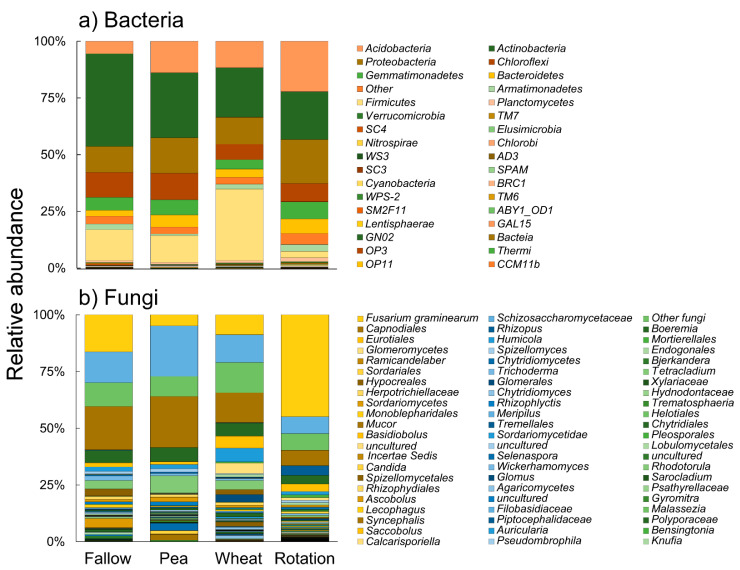
Stacked bar graphs showing the bacterial (phylum level) and fungal community (genera level) in the soil samples subject to different crop cultivation systems: fallow, pea only, wheat only, and pea–wheat rotation. Values are the average of four replicates for each treatment, with the exception of pea–wheat rotation, where ten replicates were used.

An overview of bacterial community composition at phylum level is reported in Figure 3. 

**Figure 4:**
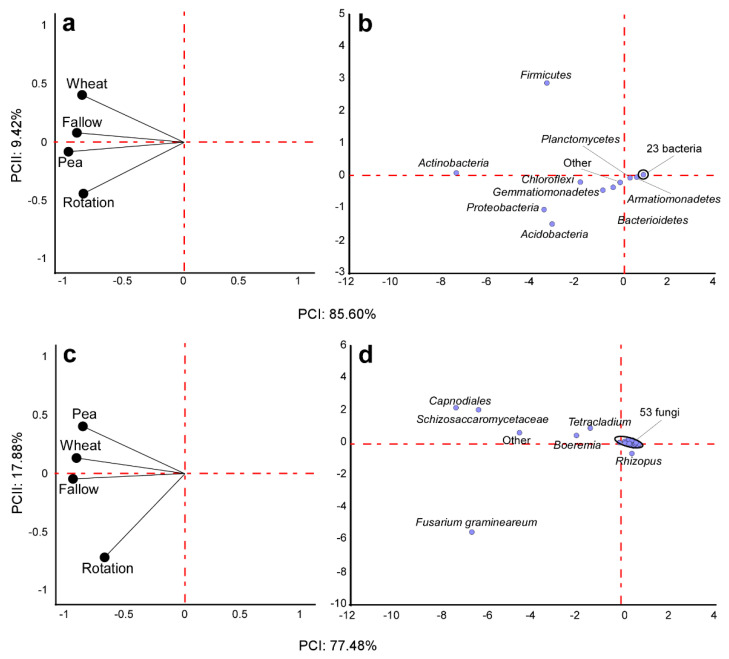
Loading (**left**) and Score (**right**) plots of principal component analysis (PCA) of the soil microbiota based on bacteria (**a**,**b**; phylum level) and fungi (**c**,**d**; genera level) as a function of the four different crop rotation management systems.

Firmicutes dominated in wheat monoculture, followed by Actinobacteria, Acidobacteria, and Proteobacteria. Pea monoculture and fallow showed similar bacterial communities with Firmicutes, which was largely substituted by Actinobacteria as the most abundant phylum. The crop rotation indicated the most distinct bacterial community, demonstrating the highest relative abundance in terms of Acidobacteria and Proteobacteria and a very minor contribution by Firmicutes. In this regard, principal component analysis (PCA) based on microbial bacterial composition at the phylum level confirmed a clear separation of the samples according to the crop management practice. The first two components accounted for 95.0% (85.6 and 9.4%, respectively) of the total variance, with samples from crop rotation and wheat monoculture appearing as the most different samples (Figure 4).

**Figure 5:**
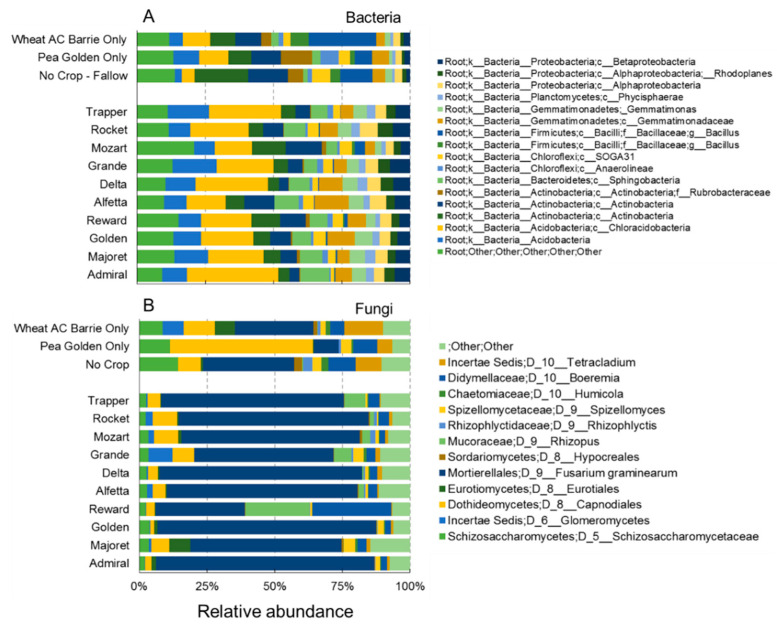
Stacked bar showing the bacterial (**A**, above) and fungal community (**B**, below) in the soil samples from wheat rhizosphere subject to AC Barrie wheat rotation with the ten pea cultivars, fallow (no crop-fallow), pea cv CDC Golden only, and wheat AC Barrie monoculture.

Considering the fungal community composition, *Fusarium*, Schizosaccharomycetaceae, Capnodiales, *Rhizopus*, and *Boeremia* were the five most common groups (Figure 3). *Fusarium graminearum* was the most abundant species in the crop rotation, with significantly lower abundance in the other crop management practices. Similarly, *Rhizopus* was abundant in the crop rotation but almost absent in the other soil treatments. *Glomus* and *Rhizophagus* were present only in the wheat monoculture and the rotation, being more abundant in the first treatment. On the contrary, *Trichoderma* showed the highest abundance in the fallow, with the lowest relative abundance in the wheat monoculture and the crop rotation. *Phoma* had the highest relative abundance in the pea monoculture, was absent in the fallow, and showed intermediate values in the wheat and crop rotation. The PCA based on fungal composition at the genus level showed a clear separation of the samples, with the first two components accounting for 95.4% (77.5 and 17.9%, respectively) of the total variance (Figure 4). Soil from the pea monoculture and the pea–wheat rotation demonstrated the greatest differences, with *F. graminearum* playing an important role in explaining this distribution pattern. Finally, soil fungal communities in the wheat monoculture and the no-crop fallow appeared relatively similar when compared to those in other crop management practices. The composition of these microorganism communities was further analyzed in the rotation between the cereal and the legume crop to observe the distributions associated with the ten different pea cultivars (Figure 5). In this more detailed analysis, *F. graminearum* was clearly noted as the dominant OTU in nine out of the ten rotations where wheat was planted in the stubble of the diverse pea cultivars. The lowest abundance of the wheat pathogen was only noted in the case of the Reward cultivar, which recorded a substantial presence of *Rhizopus*. Concerning the bacteria, Acidobacteria and Actinobacteria were the most abundant phyla in all the pea–wheat rotations (Figure 5).

## 4. Discussion

Soil can be considered as a bioreactor where complex microbiota, often composed of hundreds of co-existing bacterial and fungal species, compete for organic carbon resources and differentiate their ecological niches along with various temperature, available oxygen, and pH gradients [25]. Specifically, organic carbon sources mostly composed of root exudates and crop residues, including the turnover of fine roots, shape the microbiome diversity and composition in soil. Here, the results indicated that different crop rotations and fallow farming systems significantly modified the bulk soil microbial community composition, as well as the bacterial and fungal diversity. Notably, it was found that the crop rotations, including the planting of pea (10 different varieties) followed by seeding of wheat (a single variety) in the legume stubble in the subsequent year, reduced fungal and bacterial diversity but enhanced some pathotrophs, including *F. graminearum*, resulting in potential important implications for wheat management.

To date, neither positive nor negative responses of microbiota diversity to crop rotation have been reported in different agricultural systems [26]. In the context of the Canadian prairies, recent studies have reported that the inclusion of a pulse crop in the rotation with wheat can largely affect the rhizosphere composition of the bacterial [27] or the fungal community [17]. In another cultivation system, Niu et al. in 2018 [21] reported that rotations, continuously between pulse crops, i.e., pea and lentil, reduced rhizosphere fungal diversity and uniformity. It is well established that the rhizosphere microbiota can be actively selected by the root exudates produced by the host plant, based on the quantity and the chemical composition, which will influence the presence and the structure of the microbes associated [28]. Here, it was found that the cultivation of pea alone dramatically reduced both bacterial and fungal diversity in the soil samples in comparison to those originating from the wheat, pea–wheat rotation, and fallow conditions. This suggests that pea and other pulse crops may cause a strong microbial selection, resulting in a reduction in the fungal diversity that was limited not only to the rhizosphere but also extended outside to the surrounding soil zone.

This effect may be determined by the chemical quality of the organic carbon inputs to the soil, such as those originating from the vegetative debris of the cultivated crop, largely consisting of freshly fallen leaf litter, roots with their residual exudates, and fine root turnover, which play a prominent role in nutrient availability. For instance, in the fallow cultivation system, the microbiome could be starved in terms of limited organic carbon accessibility, due to the absence of plant material, a factor that could reduce the dominance of copiotrophic species, shifting the microbiological balance indirectly thus altering the species coexistence. In contrast, in the pulse–wheat rotation, the greater diversity of plant residues in the organic mixture could correspondingly promote the diversification of the saprotrophic microbiota present [29]. Moreover, the chemistry of the plant residues can vary dramatically depending upon the plant life form, whereby the most notable differences can be observed between nitrogen-fixing plants, such as legumes, which are rich in nitrogen, compared to graminoid plants with labile organic carbon compounds, which have a higher cellulose and lignin content and C/N ratio [30]). In general, crop residues with high nitrogen, combined with those having a low lignin content, decompose at a faster rate, causing a profound and specific selection of saprotrophic microbiota [31]. Considering that crop residues and root turnover are the primary source of organic carbon for microbiota, a better understanding of the role that crop rotation plays in terms of plant tissue chemistry would be a crucial step in developing reliable guidelines for correct crop rotation management. Further studies are also required to determine the implications of the reduced microbial diversity caused by pea on the subsequent growth and yield of wheat.

The crop rotation systems had a strong impact on the fungal plant pathogens, whereby the soil samples from all the pea–wheat rotations were substantially enriched by an abundance of *F. graminearum* in comparison to the fallow system, as well as the wheat monoculture. Notably, there was little differences observed on the effect of the ten different pea varieties in determining the presence of the pathogen found in the soils collected from the subsequent cereal planting, indicating a robust effect. *F. graminearum*, also known as Fusarium head blight [32], is considered a common pathogen of wheat grown in North America and the Canadian prairies, and together with other *Fusarium* species, including *F. redolens*, *F. tricinctum*, *F. solani*, *F. avenaceum*, and *F. oxysporum*, this fungal complex causes notable economic losses and is responsible for the production of many mycotoxins [17,33,34]. Niu et al. [21] reported that the pea rhizosphere was enriched with pathotrophic fungi, including *Fusarium* spp., especially when pulse crops were more frequently grown. *F. graminearum* abundance in the pea rhizosphere was found to have a five-fold increase when the legume was cultivated in rotation with oats. Moreover, Nayyar [35] reported that Fusarium root rot of pea significantly increased when peas were cultivated in monosuccession.

In our study, the pea–wheat rotation dramatically increased *F. graminearum* in the wheat soils, suggesting that the pea–grass rotation did not interrupt the pathogen life cycle but, instead, promoted an increase in the pathogen inoculum in the following cereal crop. At this stage, the exact causes of this effect are not known; however, it is apparent that the fungal structures remain well conserved and viable in the soil, are able to multiply in the subsequent season, then possibly infect the preferred host plant once it is again present in the environment. These results are contrary to the expected outcomes in which crop rotations with non-host plants are normally recommended as a good method of agronomic control of *F. graminearum* in order to reduce the inoculum levels [33]. This increase in the disease agent in this farming system could produce serious consequences to wheat productivity and quality. Undoubtedly, further studies are needed to understand the implications of the increased *F. graminearum* populations when pea is included in the rotation on wheat root and grain health and yield.

In comparison, it was found that the bacterial microbiota was less responsive to the crop rotation than the fungal microbiota, exhibiting fewer notable changes in this microbial composition. Previous studies have documented that pulse crops can affect the bacterial microbiota of bulk soil that are associated with the roots [36]. More recently, Hamel et al. [24] reported a decrease in Proteobacteria in crop rotations employing a frequency of high pulse crops. Overall, minor differences were noted at the phylum level in bulk soil subject to different rotations, with an increase in Actinobacteria in fallow and Firmicutes in wheat. The increase in Actinobacteria in fallow could be due to changes in soil chemistry factors such as pH or reduced availability of labile carbon forms released by the plant by exudation. In fact, Actinobacteria is considered oligotrophic, but these fungi are also capable of degrading recalcitrant carbon sources, including agrochemicals such as herbicides, fungicides, and insecticides [37]. Interesting is the notable increase in Firmicutes in the presence of wheat in the rotation. Many beneficial bacteria belonging to the Firmicutes phylum include several species that belong to the *Bacillus* genus, in which many species are noted for their capacity of biological control. This aspect deserves further investigation in order to understand their functional impact.

## 5. Conclusions

In summary, our study indicates that rotations alter soil microbial community diversity and composition in agricultural production on the Canadian prairies. In agreement with previous studies that focused on rhizospheric soil, it was found that the identity of crops grown in succession shapes the microbial composition of both the bacterial and, in particular, the fungal community. The pea monoculture was found to reduce the microbiota diversity compared to that in the rotation, as well as the fallow. However, the pea–wheat rotation increased the populations of *F. graminearum*, an important wheat pathogen. Although the impact of these microbial changes on wheat growth and yield was not assessed, this study reveals a substantial microbial shift due to the farming system implemented that warrants further functional studies to determine the causes. In addition, the frequent use of pea in crop rotations should be further evaluated and properly balanced to consider its multiple ecological effects on nitrogen mineralization, water conservation, and impact on beneficial, as well as pathotrophic microbiota.

## Data Availability

Raw DNA sequences were deposited on the Sequence Read Archive (SRA) of the National Center for Biotechnology Information (NCBI), under Accession Number PRJNA800669.

## References

[1] Tilman D., Cassman K.G., Matson P.A., Naylor R., Polasky S. (2002). Agricultural sustainability and intensive production practices. Nature.

[2] Altieri M.A. (2018). Agroecology: The Science of Sustainable Agriculture.

[3] Huang L.-F., Song L.-X., Xia X.-J., Mao W.-H., Shi K., Zhou Y.-H., Yu J.-Q. (2013). Plant-soil feedbacks and soil sickness: From mechanisms to application in agriculture. J. Chem. Ecol..

[4] Chan K., Heenan D. (1996). The influence of crop rotation on soil structure and soil physical properties under conventional tillage. Soil Tillage Res..

[5] Mäder P., Fliessbach A., Dubois D., Gunst L., Fried P., Niggli U. (2002). Soil fertility and biodiversity in organic farming. Science.

[6] Curl E.A. (1963). Control of plant diseases by crop rotation. Bot. Rev..

[7] Bailey K., Gossen B., Lafond G., Watson P., Derksen D. (2001). Effect of tillage and crop rotation on root and foliar diseases of wheat and pea in Saskatchewan from 1991 to 1998: Univariate and multivariate analyses. Can. J. Plant Sci..

[8] Peters R., Sturz A., Carter M., Sanderson J. (2003). Developing disease-suppressive soils through crop rotation and tillage management practices. Soil Tillage Res..

[9] Fernandez M.R., Fernandas J. (1990). Survival of wheat pathogens in wheat and soybean residues under conservation tillage systems in southern and central Brazil. Can. J. Plant Pathol..

[10] Xiao C., Subbarao K., Schulbach K., Koike S. (1998). Effects of crop rotation and irrigation on *Verticillium dahliae* microsclerotia in soil and wilt in cauliflower. Phytopathology.

[11] Osborne L.E., Stein J.M. (2007). Epidemiology of Fusarium head blight on small-grain cereals. Int. J. Food Microbiol..

[12] Cesarano G., Zotti M., Antignani V., Marra R., Scala F., Bonanomi G. (2017). Soil sickness and negative plant-soil feedback: A reappraisal of hypotheses. J. Plant Pathol..

[13] Miller M., Dick R.P. (1995). Thermal stability and activities of soil enzymes as influenced by crop rotations. Soil Biol. Biochem..

[14] West T.O., Post W.M. (2002). Soil organic carbon sequestration rates by tillage and crop rotation: A global data analysis. Soil Sci. Soc. Am. J..

[15] Balota E.L., Colozzi-Filho A., Andrade D.S., Dick R.P. (2003). Microbial biomass in soils under different tillage and crop rotation systems. Biol. Fertil. Soils.

[16] Lupwayi N., Rice W., Clayton G. (1998). Soil microbial diversity and community structure under wheat as influenced by tillage and crop rotation. Soil Biol. Biochem..

[17] Borrell A.N., Shi Y., Gan Y., Bainard L., Germida J., Hamel C. (2017). Fungal diversity associated with pulses and its influence on the subsequent wheat crop in the Canadian prairies. Plant Soil.

[18] Hilton S., Bennett A.J., Chandler D., Mills P., Bending G.D. (2018). Preceding crop and seasonal effects influence fungal, bacterial and nematode diversity in wheat and oilseed rape rhizosphere and soil. Appl. Soil Ecol..

[19] MacWilliam S., Wismer M., Kulshreshtha S. (2014). Life cycle and economic assessment of Western Canadian pulse systems: The inclusion of pulses in crop rotations. Agric. Syst..

[20] Gan Y., Miller P., McConkey B., Zentner R., Stevenson F., McDonald C. (2003). Influence of diverse cropping sequences on durum wheat yield and protein in the semiarid northern Great Plains. Agron. J..

[21] Niu Y., Bainard L.D., May W.E., Hossain Z., Hamel C., Gan Y. (2018). Intensified pulse rotations buildup pea rhizosphere pathogens in cereal and pulse based cropping systems. Front. Microbiol..

[22] Caporaso J.G., Kuczynski J., Stombaugh J., Bittinger K., Bushman F.D., Costello E.K., Fierer N., Peña A.G., Goodrich J.K., Gordon J.I. (2010). QIIME allows analysis of high-throughput community sequencing data. Nat. Methods.

[23] McDonald D., Price M.N., Goodrich J., Nawrocki E.P., DeSantis T.Z., Probst A., Andersen G.L., Knight R., Hugenholtz P. (2012). An improved Greengenes taxonomy with explicit ranks for ecological and evolutionary analyses of bacteria and archaea. ISME J..

[24] Quast C., Pruesse E., Yilmaz P., Gerken J., Schweer T., Yarza P., Peplies J., Glöckner F.O. (2012). The SILVA ribosomal RNA gene database project: Improved data processing and web-based tools. Nucleic Acids Res..

[25] Fierer N. (2017). Embracing the unknown: Disentangling the complexities of the soil microbiome. Nat. Rev. Microbiol..

[26] Venter Z.S., Jacobs K., Hawkins H.-J. (2016). The impact of crop rotation on soil microbial diversity: A meta-analysis. Pedobiologia.

[27] Hamel C., Gan Y., Sokolski S., Bainard L.D. (2018). High frequency cropping of pulses modifies soil nitrogen level and the rhizosphere bacterial microbiome in 4-year rotation systems of the semiarid prairie. Appl. Soil Ecol..

[28] Hu W., Strom N., Haarith D., Chen S., Bushley K.E. (2018). Mycobiome of cysts of the soybean cyst nematode under long term crop rotation. Front. Microbiol..

[29] Santschi F., Gounand I., Harvey E., Altermatt F. (2018). Leaf litter diversity and structure of microbial decomposer communities modulate litter decomposition in aquatic systems. Funct. Ecol..

[30] Cartenì F., Deslauriers A., Rossi S., Morin H., De Micco V., Mazzoleni S., Giannino F. (2018). The physiological mechanisms behind the earlywood-to-latewood transition: A process-based modeling approach. Front. Plant Sci..

[31] Bonanomi G., De Filippis F., Cesarano G., La Storia A., Zotti M., Mazzoleni S., Incerti G. (2019). Linking bacterial and eukaryotic microbiota to litter chemistry: Combining next generation sequencing with 13C CPMAS NMR spectroscopy. Soil Biol. Biochem..

[32] Schmale D., Bergstrom G. (2010). Fusarium Head Blight (FHB) or Scab.

[33] Dill-Macky R., Jones R. (2000). The effect of previous crop residues and tillage on Fusarium head blight of wheat. Plant Dis..

[34] Fernandez M.R. (2007). Fusarium populations in roots of oilseed and pulse crops grown in eastern Saskatchewan. Can. J. Plant Sci..

[35] Nayyar A., Hamel C., Lafond G., Gossen B.D., Hanson K., Germida J. (2009). Soil microbial quality associated with yield reduction in continuous-pea. Appl. Soil Ecol..

[36] Yang C., Hamel C., Gan Y., Vujanovic V. (2013). Pyrosequencing reveals how pulses influence rhizobacterial communities with feedback on wheat growth in the semiarid Prairie. Plant Soil.

[37] Schrijver A.D., Mot R.D. (1999). Degradation of pesticides by actinomycetes. Crit. Rev. Microbiol..

